# Age-Related Changes in Motor Cortical Representation and Interhemispheric Interactions: A Transcranial Magnetic Stimulation Study

**DOI:** 10.3389/fnagi.2014.00209

**Published:** 2014-08-11

**Authors:** Elisabetta Coppi, Elise Houdayer, Raffaella Chieffo, Francesca Spagnolo, Alberto Inuggi, Laura Straffi, Giancarlo Comi, Letizia Leocani

**Affiliations:** ^1^Neurological Department and Experimental Neurophysiology Unit, Institute of Experimental Neurology (INSPE), University Hospital-IRCCS San Raffaele, Milan, Italy

**Keywords:** physiological aging, transcranial magnetic stimulation, ipsilateral silent period, muscle cortical maps, motor performances

## Abstract

To better understand the physiological mechanisms responsible for the differential motor cortex functioning in aging, we used transcranial magnetic stimulation to investigate interhemispheric interactions and cortical representation of hand muscles in the early phase of physiological aging, correlating these data with participants’ motor abilities. Right-handed healthy subjects were divided into a younger group (*n* = 15, mean age 25.4 ± 1.9 years old) and an older group (*n* = 16, mean age 61.1 ± 5.1 years old). Activity of the bilateral abductor pollicis brevis (APB) and abductor digiti minimi (ADM) was recorded. Ipsilateral silent period (ISP) was measured in both APBs. Cortical maps of APB and ADM were measured bilaterally. Mirror movements (MM) were recorded during thumb abductions. Motor abilities were tested using Nine Hole Peg Test, finger tapping, and grip strength. ISP was reduced in the older group on both sides, in terms of duration (*p* = 0.025), onset (*p* = 0.029), and area (*p* = 0.008). Resting motor threshold did not differ between groups. APB and ADM maps were symmetrical in the younger group, but were reduced on the right compared to the left hemisphere in the older group (*p* = 0.008). The APB map of the right hemisphere was reduced in the older group compared to the younger (*p* = 0.021). Older subjects showed higher frequency of MM and worse motor abilities (*p* < 0.001). The reduction of right ISP area correlated significantly with the worsening of motor performances. Our results showed decreased interhemispheric interactions in the early processes of physiological aging and decreased cortical muscles representation over the non-dominant hemisphere. The decreased ISP and increased frequency of MM suggest a reduction of transcallosal inhibition. These data demonstrate that early processes of normal aging are marked by a dissociation of motor cortices, characterized, at least, by a decline of the non-dominant hemisphere, reinforcing the hypothesis of the right hemi-aging model.

## Introduction

During physiological aging, the human brain undergoes a series of morphological and functional changes. In healthy aging, cognitive and simple motor tasks produce more symmetrical and widespread cortical activation compared to young adults, due to a higher recruitment of cortical areas of both hemispheres, as supported by functional neuroimaging (Hutchinson et al., 2002; Mattay et al., 2002; Riecker et al., 2006; Ward et al., 2007) and electroencephalographic (EEG) studies (Sailer et al., 2000; Labyt et al., 2006; Vallesi et al., 2010).

In cognitive processes, this greater bilateral cortical activation seems to reflect compensatory mechanisms, in order to support cognitive abilities in healthy older people (Cabeza et al., 2002; Dolcos et al., 2002). Conversely, evidence for a positive effect of this cortical hyperactivation on motor functions is less clear. Functional neuroimaging (Riecker et al., 2006) and EEG (Inuggi et al., 2011) studies demonstrated that this bilateral cortical activation was still characterized by reduced motor abilities in aging. These results suggest that the more symmetrical motor cortex activity observed in physiological aging would not be due to a compensatory mechanism (Inuggi et al., 2011). Therefore, the functional modulations revealed in motor cortices during physiological aging are likely to be related to a loss of balance between excitatory and inhibitory circuits.

A major hypothesis is that this could be related to changes in interhemispheric connections between motor cortices (Talelli et al., 2008), due to either a loss of transcallosal fibers or to a decreased excitability of interhemispheric connections. Transcranial magnetic stimulation (TMS) has been used to investigate the effects of aging on interhemispheric inhibition (IHI). A paired-pulse TMS study, with conditioning stimulus on the hemisphere ipsilateral to the moving hand and a contralateral test stimulus, showed no age-related differences in IHI at rest, but demonstrated an enhanced IHI in young subjects during unilateral movements (from the activated hemisphere to the resting one) (Talelli et al., 2008). This IHI modulation during movement was absent in the older group, showing functional alteration of the IHI in physiological aging.

More recently, single-pulse TMS was used to study IHI in aging using the ipsilateral silent period (ISP) (Davidson and Tremblay, 2013; Petitjean and Ko, 2013). These studies demonstrated a reduction in ISP in terms of duration (Petitjean and Ko, 2013) and area (Davidson and Tremblay, 2013) in older subjects compared to younger participants. The ISP consists in stimulating one motor cortex during maximal voluntary contraction of the ipsilateral hand (Ferbert et al., 1992). The stimulated motor cortex would induce a transcallosal IHI of the opposite motor cortex (Meyer et al., 1995). This inhibition is detectable as a pause in the ipsilateral electromyographic (EMG) trace (ISP). Although both IHI and ISP represent a phenomenon of IHI, they are due to different neuronal mechanisms (Chen et al., 2003). The advantage of studying ISP is that, unlike IHI, representing a reduction of motor evoked potential (MEP) amplitude, ISP represents a direct measure of the interhemispheric control of voluntary cortical motor output (Giovannelli et al., 2009).

In line with decreased interhemispheric interactions, physiological aging can be accompanied by an increased in mirror movements (MM), or more generally motor overflow (MO), reflecting all kinds of involuntary movements that appear in other muscular districts during the execution of a voluntary muscle contraction (Hoy et al., 2004). MM are due to an impaired transcallosal inhibition (Bodwell et al., 2003) and are generally observed in neurological disorders involving the corpus callosum, such as complex congenital syndromes (Galléa et al., 2011) or also post-stroke (Chieffo et al., 2013).

The increased symmetrical motor cortex activation observed in aging could also be due to an increased cortical representation of the different muscles involved in the movement, or to an increased excitability of these motor representations. Regarding the excitability of the motor cortex in physiological aging, no consensus has been found since some authors found no significant differences in resting motor threshold (RMT) between young and older people (Pitcher et al., 2003; Oliviero et al., 2006; Fujiyama et al., 2012), while others revealed a significant increase in RMT (indicating a lower excitability) in older subjects (Rossini et al., 1992; Petitjean and Ko, 2013). Peinemann et al. (2001) found only a trend toward a slightly lower RMT in younger compared with older subjects.

Thus, in order to better understand the physiological mechanisms responsible for this differential motor cortex functioning in aging, it is necessary to provide complementary data on the different aspects of motor control. To this aim, we investigated interhemispheric interactions (using ISP) in physiological aging and correlated this functional data with cortical representation of various hand muscles (using TMS mapping) and with participants’ motor performance (using hand motor function tests). Moreover, in order to better understand the mechanisms of physiological aging, we decided to focus on the early phase of aging and included healthy people in the age-range 50–70 years old.

## Materials and Methods

### Population

Thirty-one healthy volunteers were included in this study and divided into two groups: younger (15 subjects, 6 females, mean age ± SD: 25.4 ± 1.9 years, range 21–28 years old) and older (16 subjects, 7 females, mean age ± SD: 61.1 ± 5.1 years, range 51–72 years old). All subjects were fully right-handed, having obtained the maximal score at a translated modified version of the Edinburgh Handedness Inventory (Oldfield, 1971). Participants had no history of neurologic or psychiatric disorders, drug abuse, current use of psychoactive medications, neurosurgery, and metal/electronic implants or other contraindications for the use of TMS (Rossi et al., 2009). Subjects gave their written informed consent before participating in the study, which was approved by the Institutional Ethics Committee.

### Transcranial magnetic stimulation

Transcranial magnetic stimulation was delivered by a Magstim 200 simulator (Magstim Company, Ltd., Whitland, Dyfed, UK) connected to a figure-of-eight coil (Magstim second generation, 70 mm of external diameter). EMG activity of the bilateral abductor pollicis brevis (APB) and abductor digiti minimi (ADM) muscles were recorded using surface Ag/AgCl electrodes in a belly tendon montage. The coil was positioned over the best scalp location (hotspot, marked on a fitting polyester cap that subjects were wearing) for optimal MEPs over the contralateral target muscle (APB or ADM). RMT was defined as the lowest intensity that could induce a 50 μV peak-to-peak amplitude MEP in at least 5 out of 10 trials (Rossi et al., 2009). RMTs were measured on each side. For ISPs measurements, 15 stimuli were applied at an intensity of 90% of the stimulator output (Chen et al., 2003; Trompetto et al., 2004; Spagnolo et al., 2013) while subjects were performing a voluntary maximal contraction of the ipsilateral APB muscle. In between the TMS pulses, subjects were instructed to relax for 4–8 s. Both hemispheres were tested.

For the cortical maps registration, a grid centered on the vertex was placed on the polyester cap. Intersection points of the grid lines were spaced 1 cm apart and served as visual references for coil positioning. Over each hemisphere, a total of 144 intersection points were drawn. TMS pulses were delivered at 115% RMT, starting from point 1, placed 6 cm laterally to the vertex. Then, the coil was moved following growing clockwise (for the right hemisphere) or anticlockwise (left hemisphere) spirals, from one point to another until no MEP could be evoked. Four TMS pulses were applied over each stimulating point. At the end of the session, each stimulating point as well as anatomical references (nasion, vertex, left, and right tragus) were digitized (Polhemus©, FastTrak, Colchester, VT, USA).

Electromyographic signals were sampled at 2 kHz, amplified and bandpass filtered (30–1000 Hz). Impedances were kept below 5 kΩ. Data were acquired using the SynAmp/SCAN 4.3 system (Compumedics Germany GmbH, Singen, Germany) and stored on a computer for off-line analyses.

### Hand motor performance

#### Mirror movements

Participants were comfortably seated on an armchair, their forearms and pronated hands resting on a table in front of them. They performed voluntary phasic (“brief and brisk”) thumb abductions in response to a verbal “go” command (10 trials at inter-trial interval of 4 s). EMG was recorded bilaterally. The occurrence of MM in the opposite muscles was inspected off-line. For each trial, the single rectified EMG traces were averaged. If EMG average showed an involuntary activity in the contralateral homologous muscle, MM was considered positive (MM score = 1), otherwise MM score was considered as 0 (Spagnolo et al., 2013).

#### Nine Hole Peg Test

Nine Hole Peg Test (NHPT) score (Oxford Grice et al., 2003) consisted of the time taken by the subject to insert every peg in the empty holes and then remove them and place them back in the shallow container, as quickly as possible. The test was performed twice. The fastest speed among the two trials was kept for further analyses.

#### Finger tapping

Subjects were comfortably seated on a chair, their forearm and hands resting on a table placed in front of them. Participants were instructed to press on a left-button mouse as fast as possible during 10 s with their index finger. The test was performed three times, with both hands, in random sequence. Tapping frequency was calculated using STIM software (Compumedics Germany GmbH, Singen, Germany). The mean frequency of the three trials, for each hand, was kept for analyses.

#### Martin’s vigorimeter

Grip strength was measured using the Martin’s dynamometer (Martin’s Vigorimeter; BCB Ltd., Cardiff, Wales, UK). Subjects were comfortably seated on a chair, their back leaning against the back of the chair and their feet fully resting on the floor. They were asked to grab the dynamometer with their elbow flexed at 90° and their wrist extended between 0° and 30° and squeeze as hard as they could for a few seconds. Three trials per side were recorded for each subject, and the averages of the three scores were kept for further analyses.

Hand motor function scores asymmetry was calculated by subtracting left hand (L) from the right (R) values, and normalizing by their sum:

$$\text{Asym}=\frac{\text{R}-\text{L}}{\text{R}+\text{L}}$$

### TMS data analyses

Ipsilateral silent period durations were determined by rectifying the EMG traces before average. ISP onset was defined as the latency at which the averaged EMG activity became constantly (for at least 10 ms) smaller than the averaged baseline contraction level (between −60 and −10 ms before stimulus; Spagnolo et al., 2013). ISP offset was set at the first point after ISP onset at which the EMG activity regained the baseline activity for at least 10 ms. ISP duration was defined as:

$${\text{ISP}}_{\text{duration}}={\text{ISP}}_{\text{offset}}-{\text{ISP}}_{\text{onset}}$$

We also measured the ISP_area_, which was normalized according to the degree of muscle contraction pre-trigger, in order to correct for intersubject variability. The ISP_area_ (in mV × s), was calculated as [ISP_amplitude_ × ISP_duration_], and then normalized according to the pre-trigger EMG amplitude as follows: [(ISP area − baseline area)/(baseline area × 100)]. ISP_duration_ and normalized ISP_area_ asymmetry were calculated by subtracting L from R-hand values, and normalizing by their sum:

$$\text{Asym}=\frac{\text{R}-\text{L}}{\text{R}+\text{L}}$$

To analyze the cortical maps, the four MEPs obtained at each stimulating point were averaged, and the mean peak-to-peak amplitude was measured. Then, the following parameters were calculated for each considered muscle (APB and ADM):

(1) maximal MEP amplitude;
(2) map area (total number of stimulating sites in which the mean MEP was at least 50 μV of amplitude).

### Statistical analyses

All statistical analyses were performed with SPSS/PC+ 13.0 (SPSS, Inc., Chicago, IL, USA). Normality of the data was assessed using the Kolmogorov–Smirnov test. For each variable, outliers were identified according to Tukey’s method, implemented in SPSS, and excluded from the corresponding statistical analyses. Hand motor performance scores, RMTs, ISPs, and map parameters were analyzed either with an ANOVA for repeated measures, or the Conover’s free distribution method, a non-parametric ANOVA based on ranks, depending on the data normality (Conover and Iman, 1982). For the ISP and motor performance analyses the two main factors were SIDE (two levels: right and left) and GROUP (two levels: younger and older). For the cortical maps analyses, the main factors were MUSCLE (two levels: APB and ADM) and HEMISPHERE (two levels: right and left). If a main effect or an interaction between the main factors was found, *t*-tests, Mann–Whitney, or Wilcoxon tests (for unpaired or paired data) were used for *post hoc* analyses. Asymmetry values were compared between groups using Mann–Whitney or *t*-tests, depending on the normality of the data. MM frequency was compared between groups using Chi-square analyses. After having subdivided subjects according to the occurrence of MM (including both younger and older subjects), we used non-parametric Mann–Whitney tests to confront ISP parameters between these latter subgroups (group 1: presence of MM, group 2: no MM). Cross-sectional and longitudinal correlations between TMS and behavioral data were assessed using Pearson or Spearman tests, according to the data distribution. Data were considered significant when *p* ≤ 0.05.

## Results

Eight participants (four older subjects) did not tolerate the high intensity used during ISP measurements and were thus excluded from the ISP analyses, leading thus to *n* = 12 in the older group, and *n* = 11 in the younger group for ISP and correlation analyses. However, these subjects were kept for hand motor performance and cortical map analyses.

### TMS data

Conover analysis showed no group differences in terms of RMT, but showed a significant SIDE effect (*F*_1,29_ = 13.931; *p* = 0.001), left hemisphere RMTs being lower than right hemisphere [mean RMT ± SD (% stimulator output); older group: left hemisphere = 50.9 ± 8.4, right hemisphere = 58.1 ± 12.1; younger group: left hemisphere = 48 ± 4.9, right hemisphere = 53.4 ± 11.1].

**Figure 1:**
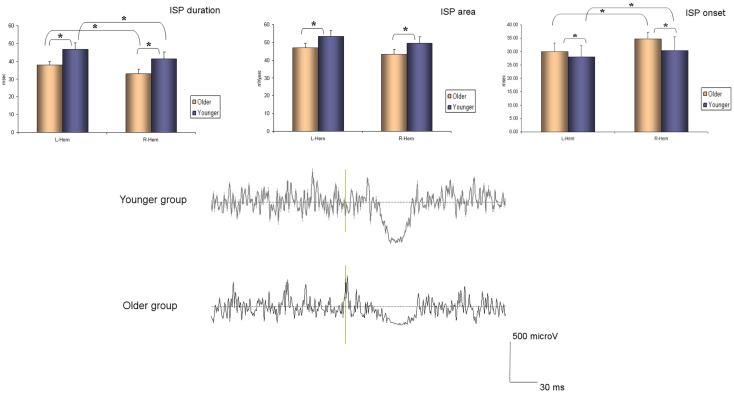
**Ipsilateral silent period parameters (duration, area, and onset) according to group and side (average ± SD)**. L-Hem, left hemisphere; R-Hem, right hemisphere. *p < 0.05. Examples of ISP traces in one older and one younger subject are exposed on the lower part of the figure.

The ANOVA for repeated measures showed a significant SIDE effect (*F*_1,18_ = 9.841; *p* = 0.006) and a significant GROUP effect (*F*_1,18_ = 5.614; *p* = 0.029) for the ISP_onset_ (one upper outlier for right ISP_onset_ in the older group and two lower outliers for right ISP_onset_ in the older group). ISP_duration_ showed a significant SIDE effect (*F*_1,21_ = 5.047; *p* = 0.036) as well as a significant GROUP effect (*F*_1,21_ = 5.798; *p* = 0.025). In ISP_area_ analyses, the ANOVA demonstrated also a significant GROUP effect (*F*_1,19_ = 8.82; *p* = 0.008), and a trend for a SIDE effect (*F*_1,19_ = 3.567; *p* = 0.074) (one upper outlier for right ISP_area_ in the older group and one lower outlier for right ISP_area_ in the younger group). Thus, in the older group ISP_onset_ was delayed, ISP_duration_ and ISP_area_ were reduced on both sides (see Figure 1). Moreover, in both groups, left hand ISP was significantly greater than right hand ISP (except for ISP_area_ where only a trend was observed). It is noteworthy that pre-trigger EMG levels were similar in both groups (*p* > 0.05). ISP data are presented in Table 1.

**Table 1:** **ISP parameters (onset, area, duration, and offset) are shown in both groups of participants (younger and older)**.

ISP parameters	Younger	*p*-Value	Older group	*p*-Value	*p*-Value
	Average ± SD	R vs. L	Average ± SD	R vs. L	Younger vs. older
Onset (ms)	**L** 27.95 ± 4.34	*p* = 0.006*	**L** 30.99 ± 3.01	*p* = 0.006*	*p* = 0.029*
	**R** 30.38 ± 5.01		**R** 35.41 ± 11.69		*p* = 0.029*
Area (mV × s)	**L** 53.28 ± 13.28	ns	**L** 46.93 ± 13.03	ns	*p* = 0.008*
	**R** 49.62 ± 11.88		**R** 43.42 ± 9.43		*p* = 0.008*
Duration (ms)	**L** 46.48 ± 11.39	*p* = 0.036*	**L** 38.05 ± 6.59	*p* = 0.036*	*p* = 0.025*
	**R** 41.5 ± 12.3		**R** 32.89 ± 9.88		*p* = 0.025*
Offset (ms)	**L** 74.63 ± 11.34	ns	**L** 69.04 ± 5.68	ns	ns
	**R** 71.88 ± 11.34		**R** 68.3 ± 11.89		ns

*L, left; R, right; SD, standard deviation; ns, non-significant*.

*Statistically significant differences.

**Figure 2:**
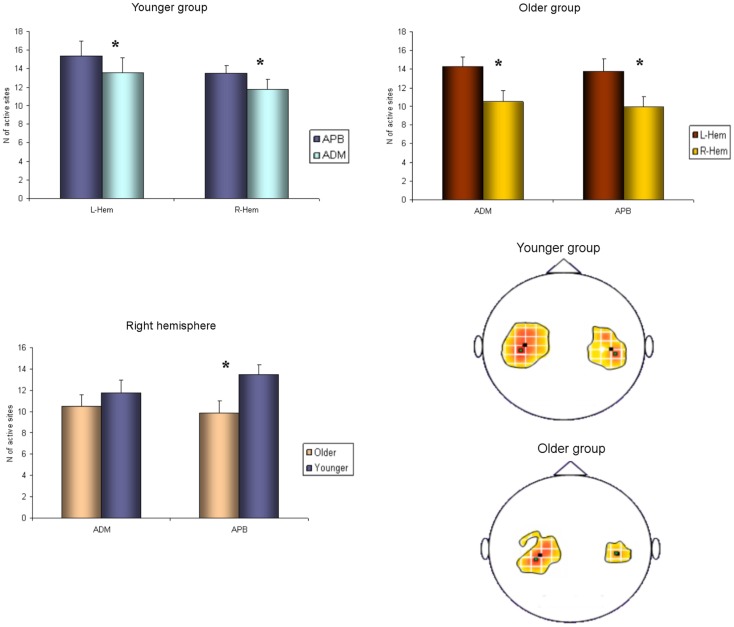
**Cortical maps of APB and ADM muscles in the younger and older groups**. Upper left graph shows representations of APB and ADM muscles in the younger group, demonstrating a significantly reduced ADM map compared to the APB, on both hemispheres. Upper right graph illustrates the significant reduction of the right hemisphere maps of both APB and ADM muscles, respected to the left hemisphere, in the older group. The lower part of Figure 2 represents the significant reduction of the APB map over the right hemisphere in the older group, compared to the younger group. *p < 0.05.

Regarding the map areas (Figure 2), in the younger group the repeated measures ANOVA showed a significant main effect of MUSCLE (*F*_1,28_ = 4.460; *p* = 0.04) and no significant main effect of HEMISPHERE (*F*_1,28_ = 1.219; *p* = 0.279), demonstrating that APB had a greater cortical representation than ADM on both hemispheres.

The older group showed no significant effect of MUSCLE (*F*_1,30_ = 0.327; *p* = 0.572), but a significant main effect of HEMISPHERE (*F*_1,30_ = 8.085; *p* = 0.008), showing that APB and ADM maps had similar areas and were reduced on the right hemisphere compared with the left hemisphere. *Post hoc* between-group *t*-test comparisons within the right hemisphere confirmed a significant reduction of APB map in the older compared to the younger group (*p* = 0.021). No such differences were observed on the left hemisphere (*p* = 0.269). For ADM maps no significant differences were found between older and younger subjects, on both hemispheres.

Conversely, the analyses of the maximal MEP amplitude (concerning both APB and ADM) did not demonstrate any significant differences, either between groups or between hemispheres (*p* > 0.05). The analyses of the asymmetry confirmed this latter result (*p* = 0.774). Cortical maps data are presented in Tables 2 and 3.

**Table 2:** **Maps parameters for APB muscles are shown in both group of participants (younger and older)**.

APB MAPS parameters	Younger group	*p*-Value	Older group	*p*-Value	*p*-Value
	Average ± SD	R vs. L	Average ± SD	R vs. L	Younger vs. older
Map area (*N* of active sites)	**L** 15.4 ± 6.17	ns	**L** 13.87 ± 5.27	*p* = 0.008*	ns
	**R** 13.53 ± 3.37		**R** 9.93 ± 4.69		*p* = 0.021*
MEP amplitude (μV)	**L** 798.77 ± 546.29	ns	**L** 819.19 ± 948.7	ns	ns
	**R** 589.18 ± 345.83		**R** 583.06 ± 652.87		ns

*APB, abductor pollicis brevis; L-Hem, left hemisphere (dominant); R-Hem, right hemisphere (non-dominant); MEP, motor evoked potential; SD, standard deviation; ns, non-significant; L, Left; R, Right*.

*Statistically significant differences.

**Table 3:** **Maps parameters for ADM muscles are shown in both group of participants (younger and older)**.

ADM MAPS parameters	Younger group	*p*-Value	Older group	*p*-Value	*p*-Value
	Average ± SD	R vs. L	Average ± SD	R vs. L	Younger vs. older
Map area (*N* of active sites)	**L** 13.66 ± 6.18	ns	**L** 14.37 ± 4.16	*p* = 0.008*	ns
	**R** 11.8 ± 4.26		**R** 10.56 ± 4.81		ns
MEP amplitude (μV)	**L** 705.38 ± 527.19	ns	**L** 1299.85 ± 1256.05	Ns	ns
	**R** 554.63 ± 330.29		**R** 631.55 ± 623.91		ns

*ADM, abductor digiti minimi; L-Hem, left hemisphere (dominant); R-Hem, right hemisphere (non-dominant); MEP, motor evoked potential; SD, standard deviation; ns, non-significant; L, Left; R, Right*.

*Statistically significant differences.

### Hand motor performance

**Figure 3:**
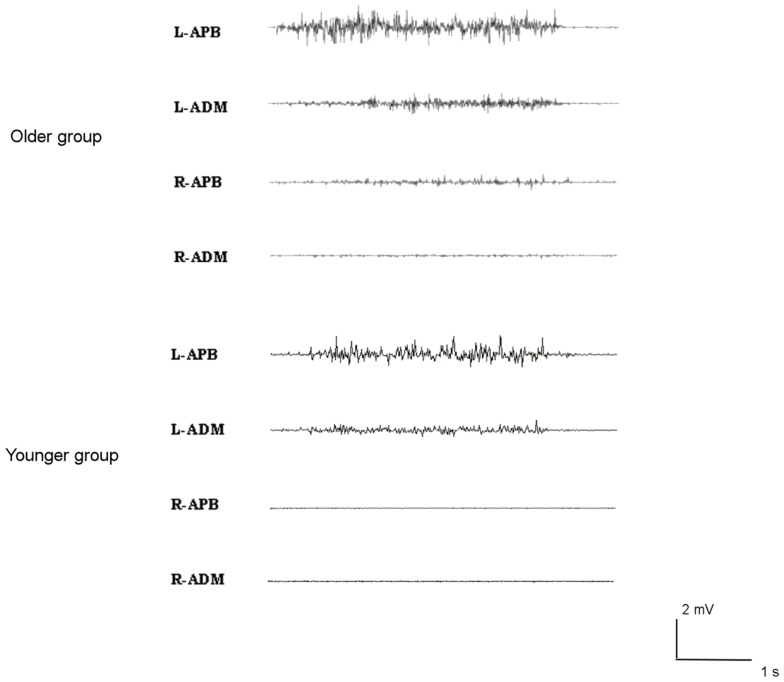
**Examples of mirror movements in an older subject (upper traces), compared to a young subject (lower traces)**.

Brisk thumb movements induced significantly more MM in the older than in the younger group (in 9 older subjects and 1 younger subject for right finger movements, *p* < 0.001; and in 13 older subjects and 5 younger subjects for left thumb extension, *p* = 0.001) (see Figure 3). Subjects showing MM did not have significantly different ISP parameters compared to subjects without MM (*p* > 0.05).

Regarding the NHPT analyses, the repeated measures ANOVA showed a significant main effect of SIDE (*F*_1,26_ = 17.219; *p* < 0.001) and GROUP (*F*_1,26_ = 29.742; *p* < 0.001), as the younger had faster scores than the older group, and the right hand scores were better than the left hand, in both groups (one upper outlier for right NHPT in the younger group, one upper outlier for left NHPT in the younger group, and one upper outlier for left NHPT in the older group). The same results were obtained for the finger tapping (FT) frequency (one upper outlier for left FT in the younger group): the younger group was faster than the older group (*F*_1,28_ = 30.337; *p* < 0.001), and performances were faster on the right compared with the left hand (*F*_1,28_ = 60.681; *p* < 0.001). The ANOVA for repeated measures showed greater grip strength in the younger group (*F*_1,29_ = 6.545; *p* = 0.016), and a strong trend for better scores with the right hand in both groups (although non-significant, *F*_1,29_ = 4.189; *p* = 0.051). The paired *t*-tests demonstrated that the asymmetrical hand motor performances (right better than left) did not differ between groups. Motor performances data are presented in Table 4.

**Table 4:** **Hand motor performances are shown in both groups of participants (younger and older)**.

Hand motor performances	Younger group	*p*-Value	Older group	*p*-Value	*p*-Value
	Average ± SD	R vs. L	Average ± SD	R vs. L	Younger vs. older
FT (Hz)	**L** 4.9 ± 0.79	*p* < 0.001*	**L** 3.65 ± 0.55	*p* < 0.001*	*p* < 0.001*
	**R** 6.44 ± 1.13		**R** 4.51 ± 0.68		*p* < 0.001*
Grip strength (kg)	**L** 0.92 ± 0.24	*p* = 0.051	**L** 0.71 ± 0.18	*p* = 0.051	*p* = 0.016*
	**R** 0.96 ± 0.26		**R** 0.73 ± 0.22		*p* = 0.016*
NHPT (s)	**L** 17.05 ± 1.62	*p* < 0.001*	**L** 20.69 ± 3.06	*p* < 0.001*	*p* < 0.001*
	**R** 15.82 ± 1.6		**R** 18.87 ± 2.14		*p* < 0.001*

*FT, finger tapping; NHPT, Nine Hole Peg Test; R-Hand, right hand; L-Hand, left hand; SD, standard deviation; L, Left; R, Right*.

*Statistically significant differences.

### Correlations

We found that hand motor performances (NHPT, FT, and grip strength) of both hands correlated significantly with the right ISP_area_: a smaller right ISP_area_ is associated with worse motor performances with both upper limbs in NHPT (for right NHPT *p* = 0.021, ρ = 0.499; for left NHPT *p* = 0.006, ρ = 0.576), FT (for right FT *p* = 0.009, ρ = −0.555; for left FT *p* = 0.027, ρ = −0.483), and grip strength (R: *p* = 0.024, ρ = −0.492; L: *p* = 0.049, ρ = −0.435). There were no further significant correlations with the other variables analyzed.

## Discussion

Our data are consistent with the fact that physiological aging is characterized by a deterioration of interhemispheric inhibition, as previously reported in recent TMS studies (Davidson and Tremblay, 2013; Petitjean and Ko, 2013). The present results bring further details on these abnormal interactions by reporting all components of ISP (duration, onset, and area) and correlating them with other measurements of motor control.

The reduction of all ISP parameters, bilaterally, reflects a declined efficiency of interhemispheric interactions, most probably due to a decreased transcallosal inhibition (Bodwell et al., 2003). Indeed, physiological aging is accompanied by a progressive degeneration of the cerebral tissue as demonstrated by the reduction in fractional anisotropy in the cerebral white matter (Pfefferbaum et al., 2000; Pfefferbaum and Sullivan, 2003; Rovaris et al., 2003; Head et al., 2004). This progressive degeneration involves also the corpus callosum, especially in its anterior portions (Abe et al., 2002; Head et al., 2004; Ota et al., 2006) that connects bilateral motor cortices (Zarei et al., 2006). The defective interhemispheric communication might thus be responsible for the more symmetrical cerebral activity observed in EEG or fMRI studies (Sailer et al., 2000; Hutchinson et al., 2002; Mattay et al., 2002; Labyt et al., 2006; Riecker et al., 2006; Ward et al., 2007; Vallesi et al., 2010) and might also be responsible for the increased frequency of MM, as previously shown in patients with agenesis of the corpus callosum (Mayston et al., 1999; Galléa et al., 2011). Indeed, our older group showed an increase in MM frequency, as the expression of a reduced ability to inhibit the tendency to activate bilateral motor cortices during a simple unimanual motor task (Cincotta and Ziemann, 2008). Thus, our results support the hypothesis of an abnormal bilateral cortical activation in the early processes of physiological aging, as described in the literature during the execution of unilateral simple motor tasks, probably due to a disruption of inter-cortical inhibitory circuits.

In our older group, the declined IHI was associated with a reduction of the cortical representation of hand muscle on the right (non-dominant) hemisphere. More specifically, while in the younger group APB and ADM muscles were symmetrically represented on the two motor cortices, in our older subjects the cortical maps resulted smaller on the right hemisphere compared with the left (dominant) one. Moreover, in older subjects, APB cortical representation on the right hemisphere was significantly reduced compared with the younger ones. No significant differences were found between groups for ADM map on the right hemisphere, probably because ADM muscle is physiologically poorly represented on motor cortices even in young people (Menon et al., 2014). As a consequence, physiological changes in hand muscles representations would be more apparent on the APB map.

This finding of a reduced cortical hand muscles representation on the right hemisphere in older subjects recalls the “right hemi-aging model” (Dolcos et al., 2002). This model is based on some evidence, especially in cognitive functions, of a progressive involution of the right cerebral hemisphere during physiological aging, but has never been demonstrated through neurophysiological studies, so far. Moreover, the hypothesis of a right hemisphere involution could explain previous findings of strong hand-dominance with a reduction of the prevalence of left-handedness in older subjects (Gilbert and Wysocki, 1992). Indeed, the progressive decline in left hand muscle representation at the cortical level could lead aging subjects to use preferentially their right hand.

We did not find significant differences in RMTs between the two groups, confirming the results of previous studies (Peinemann et al., 2001; Pitcher et al., 2003; Oliviero et al., 2006; Fujiyama et al., 2012). Thus, it seems that normal aging would not be accompanied by a change in the excitability of the pyramidal cells membrane, suggesting that Na+ and Ca2+ channels of the pyramidal cells’ axons are not modified by physiological aging (Ziemann et al., 1996). A previous study showed increased RMTs in older subjects (Rossini et al., 1992). This divergence could be explained by the higher age of the subjects (age-range 51–86 years) and the use of a different (circular) coil. Interestingly, Petitjean and Ko (2013) also found an increased RMT in the older group, but only on the right hemisphere (Petitjean and Ko, 2013). This latter result could be compatible with the “right hemi-aging model.”

As expected, the older group had slower hand motor performances. One could attribute such decline to the loss of muscle mass and strength primarily due to the loss of alpha motoneurons and the subsequent denervation of muscle fibers in aging (Doherty et al., 1993). However, these motoneuron losses are seen only after the seventh or eighth decade of age (Tomlinson and Irving, 1997). Thus, earlier changes may be attributable to supraspinal mechanisms. Indeed, Pitcher et al. (2003) demonstrated a greater trial-to-trial variability in MEP amplitudes in older subjects, attributed to a reduced synchronization of I waves in the descending volleys. Moreover, aging is associated with changes in NMDA-receptors function, in particular at the striatal level (Landfield and Pitler, 1984; Disterhoft et al., 1996; Thibault et al., 2001), leading to less efficient striatal processing of cortical information. Akopian and Walsh (2006) demonstrated age-related differences in long-term potentiation, associated with reduced sensitivity to block NMDA receptors. These modulations of corticostriatal functioning in normal aging might thus contribute to age-related deficits in striatum control of movement. These data suggest that normal aging is accompanied by impaired glutamatergic (excitatory) transmission. Moreover, inhibitory circuits would also be involved in the mechanisms of physiological aging since TMS studies demonstrated a reduced efficacy of intra-cortical inhibitory interneurons in older people. Indeed, apart from IHI deficits, age-related decreased short-interval intra-cortical inhibition (SICI) and cortical silent period durations have been shown, illustrating deficits in GABAergic (a and b) transmission (Peinemann et al., 2001; Oliviero et al., 2006; Fujiyama et al., 2012). Thus, all these previous data combined with ours suggest that early processes of physiological aging are accompanied by an imbalance in the regulation of inhibitory and excitatory circuits that might participate to the age-related worsening of motor abilities.

Degradation of motor performance in normal aging would also be related to several other factors, such as corpus callosum functions. Even unilateral movements require the functional integrity of the corpus callosum. Indeed, lesions of the corpus callosum increase the incidence of MM (Cox et al., 2012), which are also often observed in young children who have an immature corpus callosum (Mayston et al., 1999). It is thus not surprising that motor performances and ISP were significantly correlated in our study. This result might be an indirect correlation reflecting deficient corpus callosum connections and/or intrahemispheric inhibition. Indeed, ISP measurements reflect bilateral mechanisms involving activation of transcallosal fibers from one hemisphere to the other and local intra-cortical inhibitory interneurons. Thus, an ISP reduction could be due to dysfunction of one or both of these components. This could explain why right ISP area reduction correlated significantly with motor performance of both hands. If the functional decline within the right hemisphere tends to appear earlier than the left hemisphere, we may hypothesize that the right ISP measurements might be more sensitive to highlight correlations between motor performances and interhemispheric interactions. It would now be of great interest to study older aging populations in order to determine how these intra- and interhemispheric interactions evolve with time.

To conclude, our present findings suggest that early processes of physiological aging are marked by interhemispheric dissociation of motor cortices, characterized, at least in right-handed subjects, by a decline of the non-dominant hemisphere and of transcallosal interactions. In order to better qualify these early age-related changes in motor control, it would be of great interest to explore the dynamics of such changes during skilled motor behavior.

## Conflict of Interest Statement

The authors declare that the research was conducted in the absence of any commercial or financial relationships that could be construed as a potential conflict of interest.
